# One Health Approach Reveals the Absence of Methicillin-Resistant *Staphylococcus aureus* in Autochthonous Cattle and Their Environments

**DOI:** 10.3389/fmicb.2019.02735

**Published:** 2019-12-18

**Authors:** Susana Correia, Vanessa Silva, Juan García-Díez, Paula Teixeira, Kevin Pimenta, José E. Pereira, Soraia Oliveira, Jaqueline Rocha, Célia M. Manaia, Gilberto Igrejas, Patrícia Poeta

**Affiliations:** ^1^Microbiology and Antibiotic Resistance Team (MicroART), Department of Veterinary Sciences, University of Trás-os-Montes and Alto Douro (UTAD), Vila Real, Portugal; ^2^Functional Genomics and Proteomics Unit, University of Trás-os-Montes and Alto Douro (UTAD), Vila Real, Portugal; ^3^Department of Genetics and Biotechnology, University of Trás-os-Montes and Alto Douro (UTAD), Vila Real, Portugal; ^4^Faculty of Science and Technology, LAQV-REQUIMTE, University Nova of Lisbon (FCT-UNL), Lisbon, Portugal; ^5^Department of Veterinary Sciences, Animal and Veterinary Research Centre (CECAV), University of Trás-os-Montes and Alto Douro (UTAD), Vila Real, Portugal; ^6^Associação de Criadores do Maronês (ACM), Cooperativa Agrícola de Vila Real, Vila Real, Portugal; ^7^CBQF - Centro de Biotecnologia e Química Fina, Laboratório Associado, Escola Superior de Biotecnologia, Universidade Católica Portuguesa, Porto, Portugal

**Keywords:** antimicrobial resistance, One Health, methicillin-resistant *Staphylococcus aureus*, MRSA, autochthonous Maronesa cattle

## Abstract

Antimicrobial resistance represents one of the greatest challenges of the twenty-first century, and it is globally recognized that addressing this problem requires a concerted One Health approach involving humans, animals, and the environment. Methicillin-resistant *Staphylococcus aureus* (MRSA) currently represents a global burden; it is resistant to almost all beta-lactams and some MRSA strains are highly multiresistant. *S. aureus* infection in cattle results in major economic losses in the food industry. Moreover, cases of livestock-associated MRSA strains responsible for invasive life-threatening infections have been reported among human patients in contact with infected or colonized animals. The autochthonous Maronesa cattle breed is a threatened rustic traditional Portuguese breed of mountain cattle of high importance for the Vila Real region. It has been used for centuries as motive power in all kinds of agricultural work and also for meat production, which is its current dominant use and the main source of economic value, being the Maronesa meat commercialized with PDO - Protected Designation of Origin. This study aimed to determine the prevalence and transmission of MRSA in cattle of the Maronesa breed, through a concerted One Health approach comprising human, water, and soil samples of the animals’ handlers and environments. In a total of 195, 63, 40, and 43 cattle, human, water, and soil samples screened in selective ORSAB media supplemented with 2 mg/L oxacillin; only one human sample harbored a MRSA isolate which was ascribed to *spa*-type t9413 and to ST30, one of the most common genetic lineages associated with community-acquired MRSA. Considering the increasing reports of MRSA isolation from cattle and handlers in Europe, the absence of this major human and animal pathogen in Maronesa cattle and their production systems represents a serendipitous result, valuing this important autochthonous breed. To our knowledge, this is the first study to determine MRSA prevalence and transmission in Maronesa cattle. Through a concerted One Health approach, this study revealed that the Maronesa cattle and their surrounding environments do not represent reservoirs for Methicillin-resistant *Staphylococcus aureus*.

## Introduction

**Figure 1:**
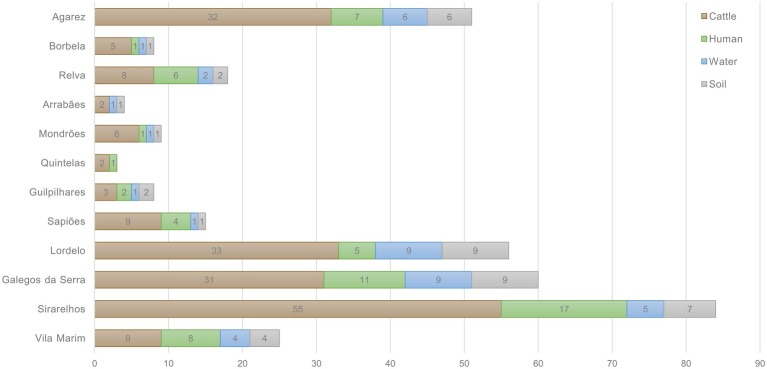
Total number of cattle, human, water, and soil samples collected from the 12 different locations in Vila Real, Portugal.

**Figure 2:**
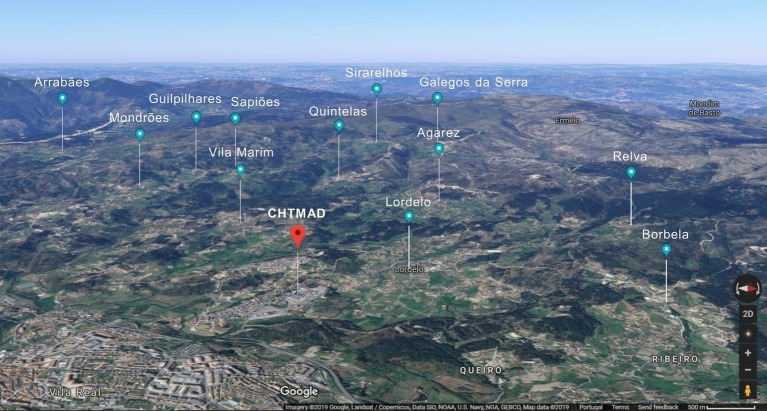
Satellite 3D image of the 12 different sampling locations in relation to the CHTMAD hospital center in Vila Real, Portugal.

Antimicrobial resistance (AMR) is one of the greatest challenges of the twenty-first century (Tacconelli and Pezzani, 2019). According to the World Health Organization (WHO), addressing the rising threat of AMR requires a holistic and multisectoral One Health approach involving humans, animals, and the environment since resistant bacteria may spread from one to the other, without recognizing human-animal or geographic borders. *Staphylococcus aureus* is a robust and versatile opportunistic pathogen that can survive in a diversity of environments (Sergelidis and Angelidis, 2017). It is frequently present in the natural flora of the nose and skin of both humans and animals, being also isolated from foods, food production systems and the environment, causing a range of illnesses from minor skin infections and food poisoning to life-threatening diseases (Cuny et al., 2015; Dweba et al., 2018; Gajdács, 2019). Methicillin-resistant *Staphylococcus aureus* (MRSA) is considered the first representative of multidrug-resistant bacteria and currently represents a global burden since it is resistant to almost all beta-lactams and can also show resistance to other major antimicrobial classes such as the fluoroquinolones (Gajdács, 2019). After being well established in the healthcare setting, MRSA has emerged in the community and subsequently in animals and food of animal origin, revealing new reservoirs for MRSA (Aires-de-Sousa, 2017; Boswihi and Udo, 2018). *S. aureus* is currently a leading cause of infection of livestock such as cows, resulting in major economic losses in the food industry (Spoor et al., 2013; Aires-de-Sousa, 2017; Boswihi and Udo, 2018). Cases of livestock-associated MRSA (LA-MRSA) strains have also been reported among human patients in contact with infected or colonized animals, which is the major risk factor for LA-MRSA colonization, being the causing agent of invasive infections in humans such as endocarditis, osteomyelitis, and ventilator-associated pneumonia (Hetem et al., 2013; Goerge et al., 2017; Boswihi and Udo, 2018). The autochthonous Maronesa cattle breed is a threatened rustic traditional Portuguese breed of mountain cattle of high importance for the Vila Real region that has been used for centuries as the motive power par excellence in all kinds of agricultural work (García, 2000). In parallel, this breed has always been used for meat production, which is its current dominant use and the main source of economic value, being the Maronesa meat commercialized with PDO - Protected Designation of Origin (García, 2000; DGAV, 2013). This study aimed to determine the prevalence and transmission of MRSA in cattle of the autochthonous Maronesa breed, through a concerted One Health approach comprising human, water, and soil samples of the animals’ handlers and environments. From February to April 2019, a total of 195 and 63 mouth and nose swabs were collected from healthy cows and their handlers, respectively. Additionally, 40 samples were collected from the cattle’s drinking water and 43 soil samples were obtained from the animals’ surroundings. Sampling was performed in 12 different locations in a radius of approximately 6 km from the CHTMAD hospital center in Vila Real, Portugal (Figures 1, 2). Mouth and nose swabs were recovered into Stuart transport media; water samples were collected into 500 ml PET flasks with sodium thiosulphate (60 mg/L); and soil was gathered into zipper seal sample bags. Samples were processed on the same day or stored at 4°C for a maximum of 24 h. Mouth and nose swabs and soil samples were incubated into Brain Heart Infusion broth containing 6.5% (w/v) NaCl for 48 h at 37°C and after seeded on Oxacillin Resistance Screening Agar Base (ORSAB) supplemented with 2 mg/L oxacillin and 50,000 IU/L of Polymyxin B. Water samples were filtrated through 47 mm 0.2 μm filters that were further placed on ORSAB plates with 2 mg/L oxacillin and 50,000 IU/L of polymyxin B. All plates were incubated for 24–48 h at 37°C and after screened for presumptive MRSA colonies. Quality of sampling was assured by observed growth of non-MRSA colonies on ORSAB and simultaneously cultured Mannitol Salt Agar plates. Confirmation of presumptive MRSA isolates was carried out by multiplex PCR of the 16S rDNA, *nuc* and *mec*A genes. Characterization by spa-typing and multilocus sequence typing (MLST) was performed using specific primers and compared with the Ridom SpaServer^1^ and MLST^2^ databases, respectively (Silva et al., 2019).

## Discussion

MRSA strains have been shown to colonize and infect a wide range of species such as livestock, companion animals, and wildlife; cases with particularly significant economic impact include bovine mastitis, poultry lameness, and severe and lethal infections in farmed rabbits (Bardiau et al., 2013; Paterson et al., 2014; Aires-de-Sousa, 2017; Gopal and Divya, 2017). Besides the importance from an animal welfare and economic perspective, MRSA in animals can also act as a reservoir for zoonotic infection of humans (Bardiau et al., 2013; Paterson et al., 2014; Aires-de-Sousa, 2017). Particularly, the abundance of clonal complex (CC)398 among livestock (mostly in mainland Europe), and the infection of humans which are often in close contact with these animals, such as farmers or veterinarians, has led to the recognition of LA-MRSA as a new MRSA epidemiological form (Bardiau et al., 2013; Paterson et al., 2014; Aires-de-Sousa, 2017). Some MRSA lineages described in cattle including CC130, CC1943, and ST425 are thought to be bovine-specific; however, certain animal lineages have been shown by molecular typing to be able to colonize or infect a wide variety of animals and also humans (Bardiau et al., 2013; Aires-de-Sousa, 2017). Moreover, the *mecC* gene, a new *mecA* gene homolog conferring methicillin resistance in *S. aureus*, has been described among bovine and human isolates and *mecC* MRSA strains reported to date are referred to mostly belong to common cattle lineages suggesting a zoonotic reservoir (Paterson et al., 2014; Aires-de-Sousa, 2017). Reports of *mecC* MRSA are presently uncommon and have been restricted to Europe; however, *mecC* MRSA represent a potential diagnostic problem due to the reliance on *mecA* or PBP2a/2′ detection for MRSA diagnosis (Paterson et al., 2014). Hence, livestock and livestock production systems have been reported to act as potential reservoirs for the emergence of new MRSA clones with the capacity to cross the species barrier and endure host-adaptive evolution, showing potential to become established in human populations worldwide as successful epidemic lineages (Spoor et al., 2013; Dweba et al., 2018). However, among the 195 cattle, 63 human and 83 environmental samples recovered from the 12 different farms included in this study and only one human sample was positive for presumptive MRSA colonies. This MRSA isolate harbored the *nuc* and *mecA* genes, confirming the *Staphylococcus* species and methicillin resistance and was ascribed to ST30 and *spa*-type t9413. ST30 is one of the most common genetic lineages associated with community-acquired MRSA (Ramundo et al., 2016) and ST30 associated with methicillin-susceptible *S. aureus* (MSSA) was one of the most prevalent clones circulating in the hospital and community in Portugal between 1992 and 2011 (Conceição et al., 2017). As far as we know, the ST30-MRSA clone has only been found among livestock animals in Portugal in healthy pigs (Pomba et al., 2009) and the presence of *spa*-type t9413 was only previously reported associated with ST22 in a comparative genomic analysis performed on food-borne *S. aureus* CC30 strains from Russia (Abaev et al., 2017). Hence, the MRSA strain detected is common among human isolates which alienates the possibility of being transmitted from cattle, reinforcing the safety of the Maronesa breed from a zoonotic point of view. Moreover, considering the increasing reports of MRSA isolation from cattle and handlers in Europe (Paterson et al., 2014; Aires-de-Sousa, 2017; Goerge et al., 2017), the absence of this major human and animal pathogen in Maronesa cattle and their production systems represents a serendipitous result, valuing this important autochthonous breed. Although the closeness to the main hospital center of the region (which according to the 2018 activity report has a MRSA prevalence of about 30%, with nearly 50% MRSA in the total *S. aureus* isolates recovered), samples were collected in higher mountain rural areas from extensive production systems that mainly use natural resources (García, 2000) and do not routinely use antimicrobials in subtherapeutic doses. This would result in lower levels of antibiotic pressure selecting for MRSA. Cattle management in the study area is characterized by farms with low head number, usually operated by both women and men, with over 55 years old on average, that work together, indicating family-type management with low economic profit. Veterinary management is scarce due to lack of literacy and low profit thus neither prophylactic treatments (e.g., deworming or vaccination schedules) nor biosecurity measures are usually implemented. Contact with the veterinarian is scarce and usually associated with the compulsory surveillance of bovine tuberculosis and brucellosis. In the case of sick cattle, assistance of the veterinarian occurs only when requested by the farmer. Antimicrobials such as procaine benzylpenicillin plus dihidroestreptomicin, ceftiofur, flofenicol and tulathromycin are administered to Maronesa cattle in cases of bovine respiratory disease (BRD), the most common disease affecting this breed. However, a lower prevalence of BRD is observed when compared to other fattening cattle breeds such as crossbreed, Charolais or Limousine since the Maronesa breed is well adapted to the local environment (extensive grazing at the mountain) (Diez et al., 2015). To our knowledge, this is the first study to determine the prevalence and transmission of MRSA in Maronesa cattle. Through a concerted One Health approach, this study revealed that the Maronesa cattle and their surrounding environments do not represent reservoirs for methicillin-resistant *Staphylococcus aureus*. Nonetheless, it would still be interesting to study the prevalence and transmission of different lineages of methicillin-susceptible *S. aureus* which can possibly evolve to MRSA and also carry resistance determinants for other major antimicrobial drug classes. Moreover, it would also be interesting to extend the study to other bacterial species that also represent major AMR threats such as carbapenem-resistant *Enterobacteriaceae* (CRE), which have a significant prevalence in the main hospital center of the region and have been increasingly detected from environmental, food, and animal sources, including cattle (European Centre for Disease Prevention and Control, 2018). It would also be noteworthy to develop similar studies in other different autochthonous breeds from extensive production systems in order to observe if similar results occur, which would give valuable insights that could lead to the implementation of new practices and policies to tackle AMR. In our perspective, many studies may have been performed with similar results that are kept undivulged due to the absence of antimicrobial-resistant strains detected and, from our point of view, the current publication principles, and incentives should encourage more the divulgation of such studies.

## Data Availability Statement

All datasets generated for this study are included in the article/supplementary material.

## Ethics Statement

This work was approved Institutionally by University of Trás-os-Montes and Alto Douro Board and written informed consent was obtained from all subjects.

## Author Contributions

SC, JG-D, KP, VS, and SO carried out the sample collection. SC, VS, KP, and SO developed the microbiology work. PT and JG-D were responsible for the collaboration that allowed sample recovery and provided information regarding the Maronesa cattle. VS performed MRSA confirmation experiments. CM and JR carried out the MLSA and *spa*-type analyses. SC wrote the manuscript. JP, GI, and PP provided critical feedback in shaping the research and manuscript.

### Conflict of Interest

The authors declare that the research was conducted in the absence of any commercial or financial relationships that could be construed as a potential conflict of interest.

## Footnotes

^1^http://spaserver.ridom.de

^2^http://saureus.mlst.net
